# Roles of physical exercise-induced MiR-126 in cardiovascular health of type 2 diabetes

**DOI:** 10.1186/s13098-022-00942-6

**Published:** 2022-11-14

**Authors:** Yixiao Ma, Hua Liu, Yong Wang, Junjie Xuan, Xing Gao, Huixian Ding, Chunlian Ma, Yanfang Chen, Yi Yang

**Affiliations:** 1 Graduate School, Wuhan Sports University, Wuhan, 430079 China; 2 Laboratory of Physical Fitness Monitoring & Chronic Disease Intervention, Wuhan Sports University, Wuhan, 430079 China; 3 Department of Pharmacology & Toxicology, Boonshoft School of Medicine, Wright State University, Dayton, OH 45435 USA; 4 Hubei Key Laboratory of Exercise Training and Monitoring, Wuhan Sports University, Wuhan, 430079 China

**Keywords:** T2DM, Exercise, MiR-126, Angiogenesis, Autophagy, Glycogenesis

## Abstract

Although physical activity is widely recommended for preventing and treating cardiovascular complications of type 2 diabetes mellitus (T2DM), the underlying mechanisms remain unknown. MicroRNA-126 (miR-126) is an angiogenetic regulator abundant in endothelial cells (ECs) and endothelial progenitor cells (EPCs). It is primarily involved in angiogenesis, inflammation and apoptosis for cardiovascular protection. According to recent studies, the levels of miR-126 in the myocardium and circulation are affected by exercise protocol. High-intensity interval training (HIIT) or moderate-and high-intensity aerobic exercise, whether acute or chronic, can increase circulating miR-126 in healthy adults. Chronic aerobic exercise can effectively rescue the reduction of myocardial and circulating miR-126 and vascular endothelial growth factor (VEGF) in diabetic mice against diabetic vascular injury. Resistance exercise can raise circulating VEGF levels, but it may have a little influence on circulating miR-126. The Several targets of miR-126 have been suggested for cardiovascular fitness, such as sprouty-related EVH1 domain-containing protein 1 (SPRED1), phosphoinositide-3-kinase regulatory subunit 2 (PIK3R2), vascular cell adhesion molecule 1 (VCAM1), high-mobility group box 1 (HMGB1), and tumor necrosis factor receptor-associated factor 7 (TRAF7). Here, we present a comprehensive review of the roles of miR-126 and its downstream proteins as exercise mechanisms, and propose that miR-126 can be applied as an exercise indicator for cardiovascular prescriptions and as a preventive or therapeutic target for cardiovascular complications in T2DM.

## Introduction

Type 2 diabetes mellitus (T2DM) is a metabolic disorder defined by serum glucose concentrations. The underlying pathophysiology is related to insulin resistance (IR), and β cell failure is required for diabetes to develop. It is characterized by hyperglycemia or hyperinsulinemia which would cause macrovascular and microvascular complications [1]. Epidemiological data have shown that cardiovascular disease (CVD) affects approximately 32.2% of patients with T2DM, accounting for over half of all fatalities recognized as a primary cause of mortality in them [2]. Glycemic control can decrease the glycated hemoglobin value and reduce macrovascular risks in patients with T1DM [3]. It seems that intensified glycemic therapy may reduce CVD risk in younger patients with recent-onset T2DM but not in high-risk older individuals with established disease [4]. A meta-analysis of data even shows that intensive glucose-lowering treatment has no benefits on death from cardiovascular causes in patients with T2DM [5]. Consequently, physical exercise as one of the first management strategies has been recommended to improve glycometabolism for cardiovascular benefits [6].

In one survey for over eight years, people with diabetes who walk at least 2 h per week have a 34% reduction in CVD mortality compared to those who do not exercise [7]. Exercise can reduce cardiovascular inflammation, blood pressure, and glucose metabolism. It has been reported to prevent the generation of reactive oxygen species (ROS) and ischemia damage, has anti-fibrosis and anti-apoptosis capabilities on diabetic myocardial and endothelial cells [8, 9]. The American Association of Clinical Endocrinologists and American College of Endocrinology recommend that each people with T2DM needs individualized exercise prescriptions to exert the maximum impact of exercise on the cardiovascular system [10]. Nevertheless, the underlying mechanisms of exercise in diabetic CVD are poorly understood.

MiRNAs are non-coding RNAs with a length of 20 ~ 24 nucleotides that can recognize the 3'-untranslated region of mRNA and inhibit the post-transcription target. They have been recognized as chemical messengers that regulate biological processes such as cell proliferation, differentiation, and survival [11]. Dysregulation of miRNAs leads to abnormal transcription of target mRNAs, which is believed to be related to various diseases. For example, circulating miR-126 is down-regulated throughout the pathophysiological processes of T2DM [12]. MiR-126 plays an essential role in the health of ECs, since mice with its deletion exhibit severe vascular leakage, dysfunction, and hemorrhaging [13]. Interestingly, aerobic exercise can partially rescue miR-126 abundance in diabetic cardiac muscle and stimulate myocardial angiogenesis by activating the vascular endothelial growth factor (VEGF) pathway [14]. Exercise can also increase circulating endothelial progenitor cell (EPC)-exosomes and their carried miR-126 for vascular repair in patients with T2DM [15].

In this review, we summarize the impact of diabetes and exercise on the abundance of miR-126, and highlight the role of miR-126 and its downstream targets that have been confirmed to participate in angiogenesis, vascular inflammation, cardiac autophagy and endothelial apoptosis. Current evidences suggest that miR-126 can be an exercise indicator for cardiovascular health and as a preventive or therapeutic approach for cardiovascular complications of T2DM.

## Vascular pathophysiology in T2DM

**Fig. 1:**
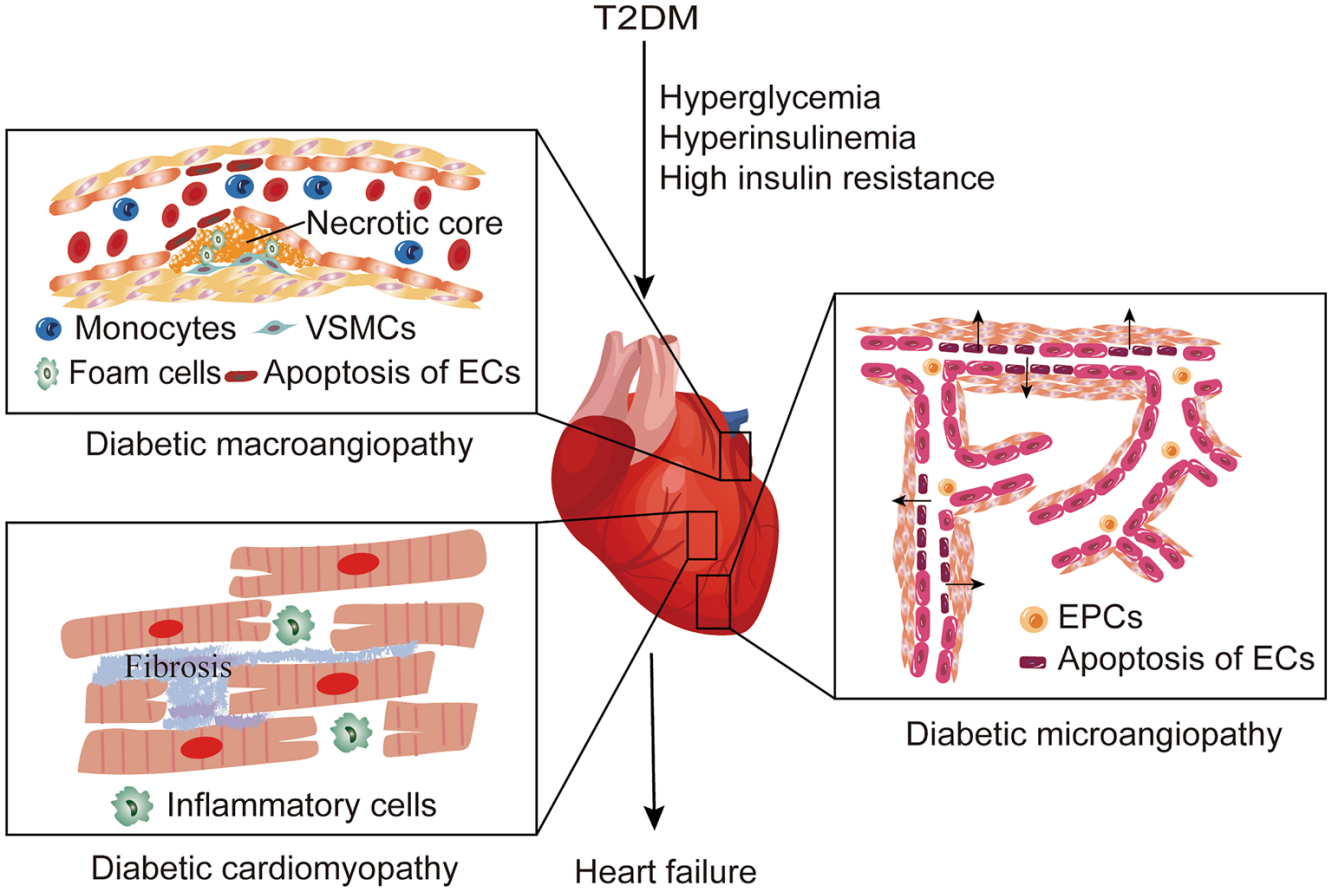
Vascular pathology in T2DM. T2DM induces macrovascular and microvascular dysfunction by hyperglycemia and hyperinsulinemia. Diabetic macroangiopathy (atherosclerosis) is made of intimal hyperplasia, foam cell infiltration, arterial wall calcification, and lumen stenosis. Capillary immature and increased permeability are features of diabetic microangiopathy. In diabetic cardiomyopathy, infiltrating inflammatory cells induce cardiac enlargement and fibrosis. All these pathological alters lead to diabetes-related heart failure. The arrow in the image means high permeability through apoptosis of ECs

Diabetic vascular disorders mainly include atherosclerosis, hypertension, and peripheral artery disease, which are developed by the abnormalities in ECs and vascular smooth muscle cells (VSMCs). ECs are located on the vasculature's inner lining for the regulation of blood flow and pressure, and the functions of leukocytes and platelets. Diabetes dramatically accelerates vascular inflammation and the formation of atherosclerotic plaques. Atherosclerotic plaque is formed by the accumulation of apoptotic ECs and macrophages, the migration and proliferation of VSMCs, and the deposits of lipid in the intima with fibrous tissue proliferation and calcinosis, which can result in severe vascular complications such as myocardial infarction or stroke [16]. The fundamental pathophysiology of diabetic macroangiopathy is depicted in Fig. 1. Furthermore, diabetes-related microvascular pathology is characterized primarily by sparse capillaries, excessive vascular permeability, and abnormal neovascularization, which eventually leads to local edema and microvascular inflammation [17, 18].

Hyperglycemia, hyperinsulinemia, and insulin resistance/insufficiency are the primary reasons of vascular injury in diabetes. Hyperglycemia inhibits endothelial NO synthase (eNOS) and induces oxidative stress through polyol, advanced glycation end products (AGEs), hexosamine, and protein kinase C pathways [19]. Hyperinsulinemia caused by IR induces reduction of the translocation of glucose transporter 4 (GLUT4), the accumulation of diacylglycerol and cellular lipids, impairment of insulin signal pathway, and metabolism problems [20]. Therefore, these pathological changes in T2DM contributes to the incidence and development of macrovascular and microvascular complications.

## MiR-126 expression in diabetic cardiovascular complications

### Decreased miR-126 as a possible cardiac biomarker of T2DM

MiR-126 located in intron 7 of the EGFL7 gene, is exclusively expressed in ECs and EPCs. It can affect the translation of endothelial-specific proteins to maintain endothelial functions. Moreover, obesity [21], diabetes [22, 23] and exercise [14, 15] have been shown to regulate the miR-126 expression. In patients with T2DM, miR-126 is an independent predictor for long-term all-cause death and likely to be an epigenetic predictor/mediator of cardiovascular complications [24]. Low level of miR-126 in plasma and in the coronary venous sinus and aorta is significantly associated with left ventricular function and cardiac repair potential in heart failure patients [25]. In addition, several logistic regression analyses indicate that hyperglycemia decreases the concentration of miR-126 in the heart [22] and plasma [23], which contributes to diabetic macroangiopathy and microangiopathy [26, 27].

The two strands of pre-miR-126 develop into miR-126-3p and miR-126-5p. Unlike several other miRNAs, the pre-miR-126 passenger strand (miR-126-5p) is not degraded and substantially has functions as the guide strand (miR-126-3p). They have the ability to identify complementary mRNA molecules and significantly trigger target mRNA degradation or translation silencing [28]. The targets of miR-126-3p predicted by Targetscan and MiRanda analysis have been verified to play a role in cardiovascular mainly including phosphoinositide-3-kinase regulatory subunit 2 (PIK3R2) [29], sprouty-related EVH1 domain-containing protein 1 (SPRED1) [30], VCAM-1 [31], and tumor necrosis factor receptor-associated factor 7 (TRAF7) [32]. The targets of miR-126-5p include high-mobility group box 1 (HMGB1) [33], activated leucocyte cell adhesion molecule (ALCAM) [34] and Notch1 inhibitor delta-like1 homolog (Dlk1) [35]. As shown in Table 1, these targets are primarily involved in angiogenesis, vascular inflammation, and EC apoptosis, which might explain why circulating low levels of miR-126 have been regarded as a biomarker for diabetic cardiovascular diseases. Although miR-126-3p has many benefits on endothelial function, it can induce atherosclerosis by increasing VSMCs proliferation via insulin receptor substrate-1 (IRS-1) inhibition [36]. IRS-1 is considered to be a risk factor for coronary artery disease [37] and its activation in the hearts of patients with T2DM induces a lower myocardial glucose utilization [38]. Additionally, miR-126-3p also suppresses target gene insulin receptor substrate-2 (IRS-2) to inhibit β cell proliferation closely correlated with the IR [39]. Thus, miR-126 and their distinct targets provide new mechanistic insights into energy metabolism and diabetic vascular complications.

**Table 1:** The target mRNAs of miR-126-3p and miR-126-5p, and their pathways and vascular functions

	Target mRNAs	Pathway	Cardiac and vascular effects	References
miR-126-3p	PIK3R2, SPRED1	PI3K-Akt-eNOS-VEGF, SPRED1-Raf-ERK-VEGF	Promoting EC growth, and migration	[29, 30]
	VCAM1, ADAM9	VCAM1-NF-κB, ADAM9-MerTK	Decreasing vascular inflammation	[31, 40]
	TRAF7	TRAF7-c-FLIP-caspases	Decreasing EC apoptosis	[32, 41]
	IRS-1	IRS-1-PI3K-Akt	Increasing VSMCs proliferation	[36]
miR-126-5p	Dlk-1	Dlk-1-Notch1-Akt-eNOS	Promoting EC growth, and migration	[35, 42]
	HMGB1, ALCAM	HMGB1-RAGE/TLR-NF-κB, ALCAM-CD6-VCAM1/ICAM1	Decreasing EC vascular inflammation	[33, 34, 43]

### EPCs deficiency in diabetic vascular complications

Circulating EPCs derived from bone marrow cells contribute to vascular repair by incorporating them into ECs monolayers and secreting vascular growth factors [44]. A low concentration of circulating EPCs defineded with CD34 + and kinase insert domain-conjugating receptor (KDR) + cells independently predicts a fourfold increase in the incidence of cardiovascular events in coronary artery disease patients [45]. Moreover, in multivariate analysis, reduced circulating EPC levels are a significant, independent predictor of poor prognosis for established cardiovascular disease [45]. Diabetes impairs both the quantity and function of circulating EPCs [46]. Anti-diabetic drug ticagrelor could significantly increase circulating EPCs in diabetic patients with non-ST elevation acute coronary syndrome [47]. Besides, EPCs isolated from obese diabetic mice significantly have reduced the ability of angiogenesis in vitro and even an anti-angiogenesis phenotype [48].

MiR-126 is abundant in EPCs and increases the proliferation, migration and tube-like structures of EPCs by targeting VEGF pathway [49, 50]. Injection of EPCs with overexpressing miR-126 can induce reendothelialization and endothelial healing by promoting extracellular signal-regulated kinase (ERK)/VEGF and Akt/eNOS signaling pathways in non-obese diabetic rats with carotid artery injury [51]. Microvesicles (MVs) released from EPCs as a kind of extracellular vehicle (EV) can modulate cell migration, apoptosis, and oxidative stress [46]. The protective effects of EPC-MVs are compromised in diabetes due to the reduction of their carried miR-126 [46]. It is acknowledged that EV is an effective way to avoid miRNAs degradation during transport. MiRNAs carried by EVs can have a variety of physiologic and pathological functions by suppressing their post-transcription of target mRNAs [52]. For example, EV can protect against acute myocardial ischemia injury by lowering cell apoptosis and increasing cell survival via carrying miR‑126 -3p by PIK3R2/VEGF signaling pathway [53, 54]. Therefore, EPCs or their EV can provide a resource of miR-126 and promote vascular repair for diabetic vascular complications.

## MiR-126 expression induced by exercise

Exercise can be classified into acute and chronic exercises according to exercise time, and aerobic and resistance exercise based on oxygen metabolism. Exhausted exercise, high-intensity interval training (HIIT), and high-, medium-, or low-intensity exercise are all classified by intensity. The current study shows that aerobic exercise is proposed to be more effective than resistance training (RT) in reducing endothelial activation markers and inflammatory cytokines [55]. Particularly, the expression of miR-126 can be comprehensively influenced by various exercise. Thus, we summarize the results of miR-126, VEGF, EPCs, and EPC-exosomes after different exercise protocols in myocardium and circulation in Table 2.

**Table 2:** Effects of different exercise protocols on the expression of circulating and myocardial miR-126, EPCs, EPC-exosomes, and VEGF

Species	Exercise intervention	Exercise Type	The changes of miR-126, EPCs, EPC-exosomes, and VEGF after exercise
Healthy individuals	A single symptom-limited exercise test	Bicycling	c-miR-126↑; [56]
Healthy individuals	HIIT: 3—min 85% of a peak power output, 4—min intervals at 40% PPO, 30 min	Bicycling	c-miR-126↑, endothelial MVs ↔ ; [57]
Healthy individuals	HIIT: 4 × 30 s all—out, 7: 30 min—intervals at 45% PPO	Bicycling	c-miR-126↑; [58]
Healthy individuals	HIIT: 95% of a peak power output, 4 × 4 min, 3—min intervals at 45% PPO	Bicycling	c-miR-126↑; [58]
Healthy individuals	HIIT: 90—95% of a peak power output, 4 × 4 min, 3—min intervals	Bicycling	c-miR-126 ↔ , c-VEGF↑; [59]
Healthy individuals	A marathon	Running	c-miR-126↑; [56, 60]
Healthy individuals	HT: 70% of the individual anaerobic threshold, 4 h	Bicycling	c-miR-126↑; [56]
Healthy individuals	MT: 60% of a peak power output, 30 min	Bicycling	c-miR-126 ↔ , endothelial MVs ↔ ; [57]
Healthy individuals	MT: 60% V̇O_2max_, 30 min	Running	EPC↑; [61]
Healthy individuals	MT: 60% of a peak power output, 90 min;	Bicycling	c-miR-126↑, c-VEGF↑; [59]
Healthy individuals	MT: 55% of a peak power output, 130 min;	Bicycling	c-miR-126↑; [58]
Healthy individuals	CE: (4—7) × 30 s, all—out, 30 s intervals at warm—up speed, 3 d/wk × 4 wks	Running	c-miR-126↑; [62]
Healthy individuals	CE: 4 × 30 s, all—out, 30 s intervals at 8.0 km/h, 2 d/wk × 4 wks	Running	c-miR-126↑; [62, 63]
Healthy individuals	CE: 74.75% of HR_max_, 25 min, 3 d/wk × 4 wks	Running	c-miR-126↑; [62]
Healthy individuals	CE: 55%—75% V̇O_2max_, 3 d/wk × 20 wks	Bicycling	c-miR-126↑; [64]
Healthy individuals	CE: marathon routine training	Running	EPC↑; [65]
Healthy individuals	3 sets × 15 repetitions of six machine resistance exercises	RT	EPC↑, c-VEGF↑; [66, 67]
Healthy individuals	3 sets × 15 repetitions of three machine resistance exercises	RT	c-miR-126 ↔ ; [56]
Healthy individuals	60%, 70% or 80% of 1RM; 12 repetitions × 4 exercises, 30 min	RT	EPC↑, c-VEGF↑; [67]
Healthy individuals	CE: 3 sets of 10 repetitions × 5, 3 d/wk × 6 wks	RT	c-VEGF↑; [68]
Healthy individuals	CE: resistance routine training	RT	mu-miR-126↓; [69]
Obese individuals	HT: 75% V̇O_2max_, 30 min	Running	c-miR-126↑; [70]
T2DM patients	MT: 60% V̇O_2max_, 30 min	Running	EPC ↔ ; [61]
T2DM patients	Strength training circuit, 30 min and walking at 60%—70% of HRR, 40 min	RT & walking	c-miR-126 ↔ ; [71]
C57BL/6 J mice	CE: 5 or 10 m/min; 60 min/d, 5 d/wk × 4 wks	Running	EPC↑, EPC-miR-126↑, EPC-exo↑, EPC-exo-miR-126↑; [15]
db/db mice	CE: 9—13 m/min, 10 × 5 min, a slope of 10°, 5 d/wk × 8 wks	Running	m-miR-126↑, m-VEGF↑, c-miR-126 ↔ ; [72]
Wistar rats	CE: 60 min/d, 5% body overload, 5 d/wk × 4 wks	Swimming	m-miR-126↑, m-VEGF↑; [73]
Wistar rats	CE: 60 min/d in 1–8 week; 120 min/d in the 9th week, 180 min/d in the 10th week; 5% body overload, 5 d/wk	Swimming	m-miR-126↑, m-VEGF↑; [73]
Wistar rats	CE: voluntary exercise, 3000 m/d, 8 wks	Running	m-miR-126↑; [74]
STZ rats	CE: 95—100% V̇O_2max_, 6d/wk × 6 wks	Running	m-miR-126↑, m-VEGF↑; [75]
STZ rats	CE: voluntary exercise, > 2000 m/d; 6 wks	Running	m-miR-126↑; [22, 76]
STZ rats	CE: 25 m/min, 30 min, a slope of 5%; 8wks	Running	m-miR-126↑, m-VEGF↑; [14]
STZ rats	CE: 10—39 m/min, 30 min, 5 d/wk × 6 wks	Running	mu-miR-126↑; [77]
Obese Zucker rats	CE: 4% of body overload, 60 min, 5 d/wk × 10 wks	Swimming	m-miR-126↑, m-VEGF↑; [78]
SHR rats	CE: 4% of body overload, 60 min, 5 d/wk × 10 wks	Swimming	c-miR-126 ↔ ; [79]
MI rats	CE: 15 m/min, 3—min × 7 & 25 m/min, 4 min × 7, 5d/wk × 4 wks	Running	m-miR-126↑; [80]

miR-126 in Table 2 refers to miR-126-3p. ↑: increased; ↓: decreased; ↔ : negligible effect; c-miR-126: circulating-miR-126; mu-miR-126: muscle-miR-126; m-miR-126: myocardial-miR-126; c-VEGF: circulating-VEGF; m-VEGF: myocardial-VEGF; HVT: high volume session; MT: Moderate training; CE: chronic exercise; HDL: high-density lipoprotein; MVs: microvesicles; EPC-exo: EPC-exosomes; SHR: spontaneously hypertensive rats; STZ rats: diabetic rats built by injection with streptozotocin; SHR: spontaneously hypertensive rats; MI: myocardial infarction

### MiR-126 expression is increased by aerobic exercise

The expression profile of circulating miRNAs provides insight into the potential exercise mechanism of the cardiovascular system, in which miR-126 is one of these miRNAs that can be directly altered by exercise intervention [81]. Based on the few studies conducted so far, chronic aerobic exercise has been demonstrated to raise circulating and myocardial miR-126 levels in both healthy and diseased people. As shown in Table 2, four weeks of high-intensity exercises can elevate the level of circulating miR-126 in healthy individuals [62, 63]. Moreover, ten weeks of moderate-and high-volume swimming in Wister rats significantly increases the level of circulating miR-126 with exercise-induced cardiac angiogenesis in a dose–response manner [73]. Chronic running increases the myocardial expression of miR-126 in STZ rats and db/db mice [22, 72], but has no impact on circulating miR-126 [72]. Likewise, there is no change of miR-126 in the hypertensive rat’s plasma after ten-week swimming, but the level of its downstream target gene protein PIK3R2 reduces from an increase of 51% to normal [79]. The comparative study on chronic exercise of diabetic mice from 8-week-old and 16-week-old indicates that early exercise intervention can increase myocardial miR-126 and VEGF, and better improve the onset and progression of diabetic heart disease [72]. Combination of exercise and diet control for six weeks in obese adolescents increases serum miR-126 and vascular endothelial diastolic functions [82]. These findings suggest that miR-126 could be the essential molecule influenced by chronic aerobic exercise in a dose–response manner and might be involved in early protecting the cardiac function.

Acute aerobic exercise is a single bout of aerobic activity that usually uses % maximal oxygen uptake (V̇O_2max_) to measure the intensity of exercise. V̇O_2max_ is the highest oxygen consumption attainable during maximal or exhaustive exercise per minutes. A single symptom-limited exercise test is a kind of exhaustion test. When a healthy subjects perform this test by bicycling, the circulating miR-126 will be increased [56]. After a 30-min aerobic exercise at 75% V̇O_2max_ in obese adults, circulating miR-126 is also increased and is continued to increase 1 h [70]. A 42-km marathon can immediately increase plasma miR-126 by 1.9-fold in trained runners, and then decline to pre-race level within 24 h [60]. Uhlemann and colleagues believed that the rise of circulating miR-126 after an acute aerobic exercise as evidence of damage of the ECs layer [56]. However, the fact is that neither mature ECs exfoliation nor ECs monolayer injury occurs after exercise, even after 30 min of acute cycling at 85% peak power [57]. Tissue hypoxia induced by high-intensity exercise might be responsible for the increase of miR-126 expression. Hypoxia induces the expression of hypoxia-inducible factor -1α (HIF-1α) and its downstream Ets-1 which can regulate miR-126 in vascular ECs [83, 84]. In addition, EPCs promoted from the bone marrow into the bloodstream might be another reason for circulating miR-126 increase. However, the circulating EPCs do not change in T2DM patients following a 30 min-treadmill exercise at 60% V̇O_2max_ [61] and increase in marathon athletes and in mice by eight week-aerobic exercise [15, 65]. Therefore, miR-126 expression/release can be different in different conditions of health, and after different protocols of aerobic exercise. It is recommended that exercise with moderate intensity or above can significantly increase circulating miR-126.

### MiR-126 expression is increased by HIIT

HIIT consists of bouts of more than 90% V̇O_2max_ followed by a brief rest period and seems to induce larger beneficial adaptation in the cardiovascular system [85]. Even a session of HIIT can completely prevent the normal postprandial reduction of endothelial function [86] and increase the circulating miR-126 levels including miR-126-3p and miR-126-5p in healthy individuals [57, 58]. In clinical experiments, the isolated high-density lipoprotein from chronic heart failure patients after moderate intensity exercise training rescues the miR-126 reduction in co-cultured ECs compared with no-exercise chronic heart failure patients [87]. In addition, the study finds that high volume training (HVT) with cycling for 130 min at 55% peak power output (PPO) could significantly elevate serum miR-126 level immediately [58]. One session of HVT with 90 min cycling at 60% PPO dramatically raises circulating miR-126 abundance in young male cyclists, but 4 × 4 min HIIT at 90–95% PPO had no influence [59]. Schmitz and colleagues also found that no acute elevation of circulating miR-126 was observed after HIIT with running 4 × 30 s at maximum speed in moderately trained students, but obviously elevated after four-week training sessions [63]. Therefore, HIIT can increase the abundance of serum miR-126, but it is influenced by training level of the subjects and the amount of exercise time.

### MiR-126 expression is affected by resistance exercise

Resistance training (RT) has remarkable cardiovascular benefits. For example, a 12-week training program consisting of moderate-intensity resistance exercise improved cardiovascular fitness in overweight and obese participants [88]. RT is recommended to improve blood pressure, reduce arterial stiffness, and benefit for glycemic control as a complement to aerobic exercise programs [89]. It has been shown that the strength training intervention for one week has no impact on the circulating miR-126 among older diabetic patients and control subjects [71]. The researchers observe that decreased miR-126 in muscle biopsies could reliably distinguish between powerlifters and controls, implying that it might help define the powerlifter phenotype [69]. Although RT does not enhance the plasma level of miR-126, it still promotes a rise in serum VEGF and the level of circulating EPCs [66, 67]. The increase of plasma VEGF and HIF-1α after anaerobic RT is probably due to muscle ischemia during exercise [67]. Eccentric training is another strength training technique which is also confirmed to cause an increase of HIF-1α in untrained skeletal muscle and to up-regulate VEGF and eNOS expressions [90]. Therefore, RT has cardiovascular and muscular effects, but its mechanism may not be associated to miR-126.

### The level of miR-126 in EPCs and EPC-EVs is increased by exercise

EPCs carried miR-126 may contribute to exercise-induced cardiovascular protection. After 12 h of a marathon, professional runners had a higher circulating percentage of EPCs which is accompanied by favorable effects on heart rate and blood pressure [65]. In diabetes patients, although circulating EPC-EVs are increased, their carried miR-126 and the expression of VEGF receptor 2(VEGFR-2) are decreased [46]. Surprisedly, exercise can significantly increase the number of circulating EPCs and EPC-exosomes and their carried miR-126 in mice, which protects ECs against from hyperglycemia-induced damage [15]. Our study showed that long-term moderate exercise mice promoted a higher circulating EPC-exosomes which could alleviate the dysfunction of injured ECs via SPRED1 downregulation and VEGF upregulation [15]. Furthermore, three years of regular endurance resistance exercise increased the circulating levels of EPCs and VEGF [66]. However, a 30-min running at 60% V̇O_2max_ could not change the number of EPCs in the impaired glucose tolerance and T2DM subjects, only conversely increases in the control group [61]. Thus, chronic exercise may provide a potential approach to reduce diabetic vascular complications by elevating the number of EPCs, EPC-EV, and its carried miR-126.

## Mechanism of exercise-induced miR-126 in improving cardiovascular function in diabetes

**Fig. 2:**
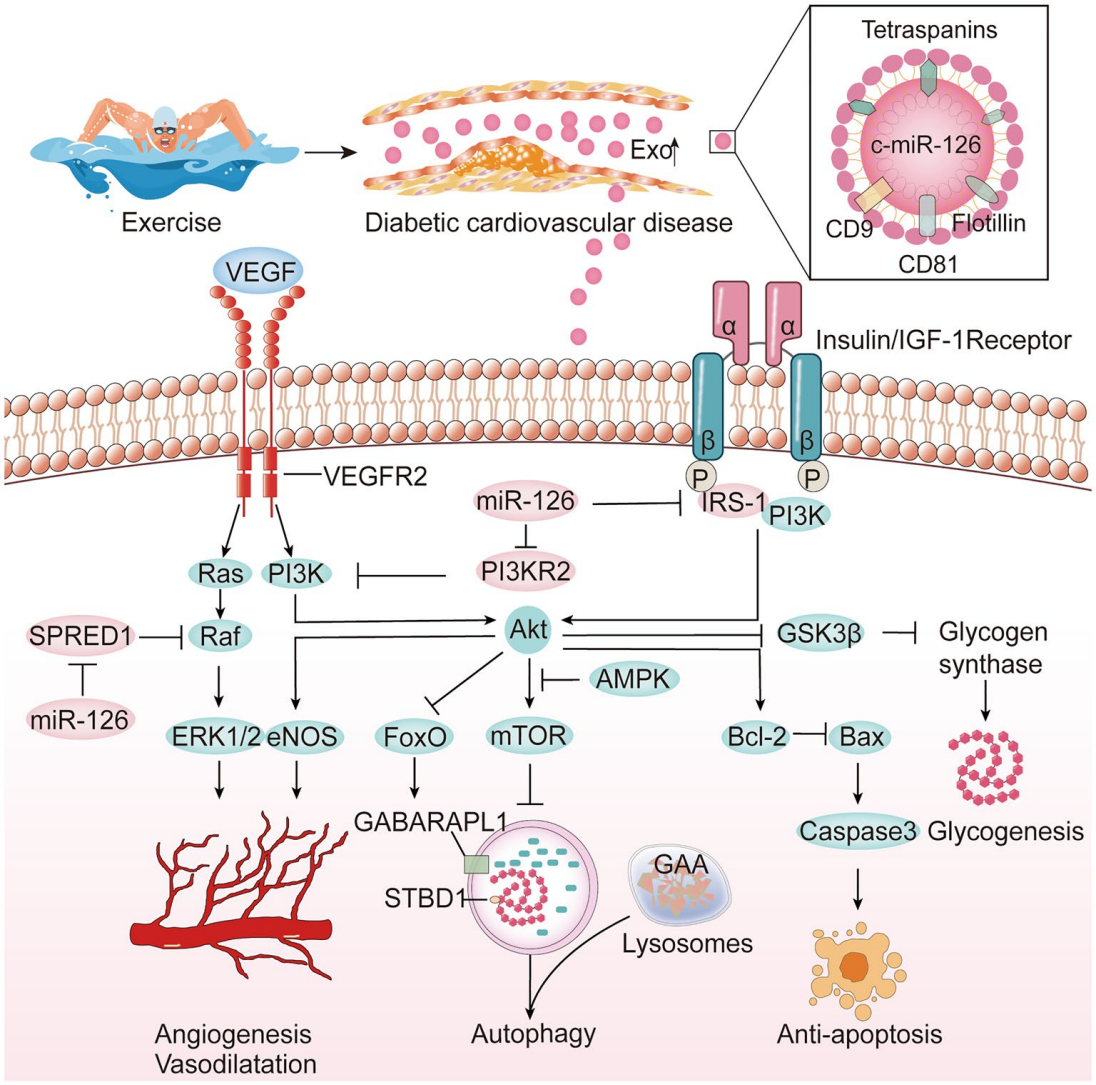
The mechanism of exercise in improving cardiovascular function in diabetes. Exosomes carried miR-126-3p/-5p plays an important role in control of angiogenesis, autophagy, anti-apoptosis, and glycogenesis, which together contribute to improve diabetic cardiovascular health in diabetes. SPRED1 and PIK3R2 suppress VEGF by separately inactivating the downstream Ras/Raf-1/ERK and PI3K/Akt signaling pathways. Akt phosphorylation is related to autophagy by affecting FoxO and mTOR, as well as anti-apoptosis by increasing Bcl-2. Akt can also phosphorylate glycogen synthase kinase-3β(GSK3β) for inactivation, which reduces inhibition of GS to increase glycogen

**Fig. 3:**
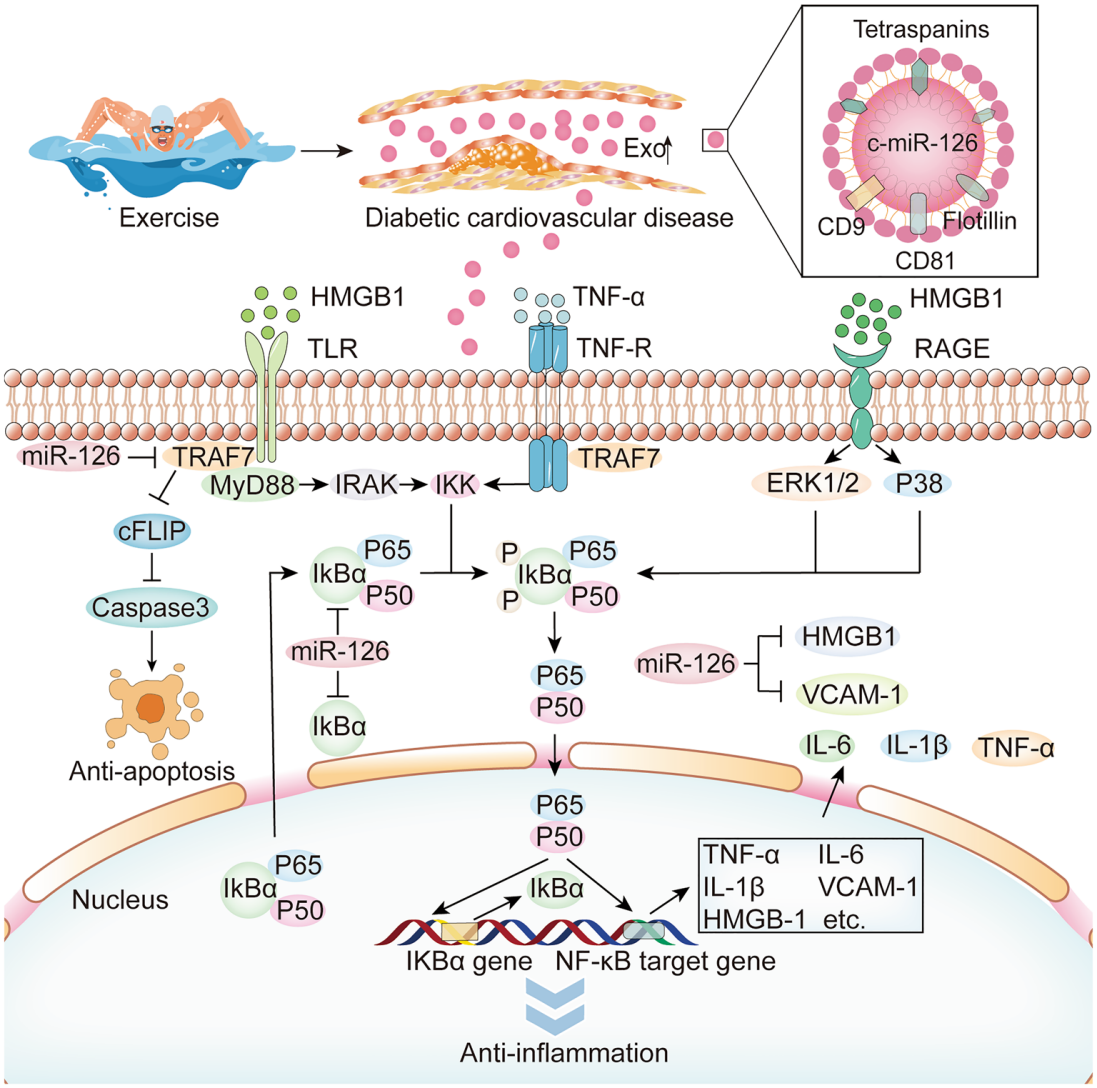
The molecular mechanisms of exercise-induced exosomal miR-126 for anti-inflammation in diabetes. Exercise-induced exosomes modify the downstream target genes IκBα, HMEB1, and VCAM-1 via their carried miR-126. The anti-inflammatory effects in diabetic cardiovascular system are achieved by inhibiting inflammatory substances, such as HMGB1 and TNF-a activate IKK to enhance phosphorylation of the NF-κB (p65 and p50) complex. Then NF-κB enters the nucleus via nuclear pore to stimulate IκBα genes and NF-κB target genes production, which can activate the NF-κB inflammatory pathway

Exercise-induced miR-126 should be a valuable marker for optimizing individual training interventions based on its biological effects. MiR-126 has multiple functions such as glucose metabolism, neovascularization, inflammatory resistance, autophagy, and anti-apoptosis effects by modulating the expressions of its target genes. Figures 2 and 3 summarize the targets of miR-126 elicited by exercise for the beneficial effects on cardiovascular health in diabetes.

### Glycometabolism

Exercise has the potential to improve glycemic control in diabetes depending on the duration and intensity of physical activity. For example, a single bout of moderate-intensity aerobic exercise reduces fasting glucose (FG) level, but less effectively than HIIT in patients with T2DM [91]. Resistance exercise can lead to a modest reductions in FG [68, 92, 93]. RT enhances glucose uptake via increasing transport of glucose into skeletal muscle by GLUT4 translocation [94, 95]. The major molecular pathways of aerobic exercise are associated with insulin sensitivity improvement which can increase skeletal muscle glucose absorption by reducing adipokines, inflammation, and oxidative stress [96]. RT can also considerably lower the inflammatory biomarker C-reactive protein, total cholesterol, and low-density lipoprotein cholesterol to increase insulin sensitivity and decrease the risk of CVD [68, 92]. The expression of miR-126 can be surprisingly increased or maintained well in circulating EPC-MVs when the plasma glucose level is well-controlled in diabetic patients by drug management [46]. It assumes that exercise has a significant influence on the increase of miR-126, which might be mediated by blood glucose regulation, but this has yet to be verified.

MiR-126 is closely associated with glucose homeostasis. The study finds that the glycogen content is much more in the placentas of miR-126−/− mice [97]. Akt activation is a critical step to phosphorylate GSK3β in glycogen synthesis process. It is evidenced that miR-126 enhances the biological function of EPCs under oxidative stress via PI3K/Akt/GSK3β signaling pathways [98]. Interestingly, there is an increase in cardiac IRS1-PI3K activity and a reduction of GLUT4 in patients with T2DM [38]. MiR-126 is a negative regulator of IRS-1 [99] and can be increased by exercise which improves insulin sensitivity and the activation of Akt in diabetic heart [15, 100]. However, exercise does not enhance IRS-1-mediated PI3K activity, but up-regulate GLUT4 protein expression in diabetic skeletal muscle [101]. Moreover, exercise can decrease [102] or increase [103] myocardial glycogen synthesis. A report showed that 13-week swimming significantly reduced high blood glucose and cardiac glycogen in Zucker diabetic fatty rats via Akt/GSK3 signaling pathway [102]. Another study found that exercise did not correct abnormal cardiac glycogen accumulation in the db/db mice [103]. The mechanism of exercise induced glycogen production is complicated in diabetic heart. The AMP-activated protein kinase (AMPK) pathway also directly controls carbohydrate metabolism. Although AMPK activation enhances cellular glucose absorption and decreases glycogen production by inhibiting glycogen synthase activity, glucose-induced allosteric activation of glycogen synthase can mask the effect of AMPK to promote glycogen accumulation, contributing to metabolic disorders [104]. Whether miR-126 participates in the exercise-related glycogen alteration requires further investigation.

### Angiogenesis

As shown in Table 1 and Fig. 2, SPRED1 and PIK3R2 are two essential miR-126-3p targets in the promotion of ECs growth and migration. SPRED1 is a member of the sprouty/spred protein family which can suppress VEGF by inactivating the downstream Ras/Raf-1/ERK signaling pathway [49]. By targeting the SPRED1 and PIK3R2, miR-126 regulates vascular endothelium permeability and angiogenesis [29]. Moreover, eNOS can be activated by PI3K/Akt/VEGF dependent pathway to control vasodilatation [105]. High glucose suppresses angiopoietin-1 (Ang-1) through the miR-126/PIK3R2 pathway in ECs [106]. This pathway not only fine-tunes VEGF-signaling on the formation of the initial vascular plexus but also strongly enhances the activity of Ang-1 on vessel stabilization and maturation. In addition, Notch1 inhibitor delta-like1 homolog (Dlk1) is the target gene of miR-126-5p that can inhibit Notch1 activation and limit G1-S phase progression to proliferation, up-regulation of miR-126-5p consequently can preventing the pathological process of atherosclerosis by inhibiting Dlk1 [35].

MiR-126/VEGF pathway is an essential exercise mechanism in diabetic cardiovascular protection. Exercise modulates miR-126 and its targets for physiological response and adaptation. Chronic and acute aerobic exercise promotes an increase in myocardial capillary/fiber ratio via miR-126/SPRED1 and PIK3R2 pathways in rats [73]. Particularly, acute HIIT can raise circulating VEGF, although its effect on circulating miR-126 is controversial [59, 62]. Therefore, based on the responses of miR-126 and VEGF, more consideration should be given to choosing appropriate intensity exercise for angiogenesis, such as HIIT or chronic aerobic exercise with moderate intensity.

### Anti-inflammation

The vascular inflammation is a crucial cause of accelerating atherosclerosis and diabetic vascular disorder. Nuclear factor-kappa B (NF-κB) plays a central role in the induction of many pro-inflammatory cytokines to regulate cell stress, cell survival, and cell proliferation. Likewise, NF-κB in diabetic heart is medicated by tumor necrosis factor-α (TNF-α), interleukin-1(IL-1), and interleukin-6 (IL-6) contributing to IR development and cardiac dysfunction [107]. Exercise has significant benefits on the reduction of oxidative stress and vascular inflammation depending on the appropriate exercise protocols. Moderate endurance exercise always exerts an anti-inflammatory effect by inhibiting pro-inflammatory cytokines [108], reducing NF-κB activation [109], limiting IR [110] and reserving mitochondrial activity [111]. Even a single session of aerobic exercise also can lower postprandial lipemia, monocytic TNF, and NF-κB activity in peripheral blood mononuclear cells against high-fat diet-induced inflammation [112, 113]. Interestingly, IκBα is a physiological inhibitor protein of NF-κB, and its phosphorylation by IκB kinase (IKK) promoting ubiquitination and active NF-κB release [114]. NF-κB p65 and NF-κB p50 can be transferred into the nucleus, stimulating IκBα and pro-inflammatory cytokines gene expressions, and then moved from the nucleus via the nuclear pore to cytoplasm by binding with IκBα. MiR-126 is an important regulatory factor in the inflammatory response, because it can directly inhibit IκBα [115]. However, the study found that IκBα protein did not alter during moderate activity but considerably was reduced by exhaustive exercise [31]. It is suggested that the increased inflammation may be closely related to exercise load.

Patients with T2DM have a high incidence rate of CVD partly due to vascular inflammation that accelerates atherosclerosis and diabetic endothelial dysfunction. Many inflammatory mediators are involved in this process, such as increased IL-6, VCAM-1, and monocyte chemoattractant protein [116]. As the downstream target of NF-κB, VCAM-1 targeted by miR-126-3p can be up-regulated by hyperinsulinemia [117, 118]. Aerobic exercise training can reduce soluble VCAM-1 and decrease leukocyte adherence [119]. In addition, miR-126 also controls the sirtuin 1 (SIRT1) and superoxide dismutase-2 expression against ROS imbalance in hind limb ischemia-subjected ob/ob mice [120]. Exercises decrease ROS production and increase NO availability in hypertensive patients [121] and the diabetic patients [122]. Inhibiting miR-126 increases the inflammatory markers and ROS generation in EPCs cultured under hyperglycemic conditions by PI3K/Akt pathway [123], which is crucial for modulating EPC migration and proliferation in T2DM [49, 123].

As a nuclear non-histone DNA-binding protein, HMGB1 is suppressed by miR-126-5p. HMGB1 level is increased in the serum of hyperglycemic rats and the management of hyperglycemia with insulin might decrease serum HMGB1 level [124]. HMGB1 could up-regulate toll-like receptors (TLR) [125] and receptor for advanced glycation end-products (RAGE) to enhance proinflammatory activities [126]. A tight junction complex composed of Claudin, Occluding, and ZO-1 is down-regulated potentially by the HMGB1/RAGE/Erk signaling pathway in the differentiated epithelium [126]. Overexpressing miR-126 in ECs under hyperglycemic condition could decrease inflammatory cytokines including TNF-α, ROS, and NADPH oxidase activity by inhibiting HMGB1 [33]. Human umbilical cord mesenchymal stem cell-derived exosomes carried overexpressing miR-126 alleviate the hyperglycemia-induced inflammation by reducing HMGB1 in human retinal ECs [127]. Moreover, HMGB1 is negatively related to post-exercise heart rate recovery in post-infarction patients, indicating that HMGB1 was involved in autonomic dysfunction during exercise [128]. As shown in Fig. 3, exercise decreases inflammation and improves diabetic cardiovascular function, probably through the miR-126-5p/HMGB1 pathway.

### Autophagy

Autophagy is a critical defense mechanism for myocardial cell protection. It plays an important role in maintaining homeostasis by restoring or eliminating damaged organelles and lipids. MiR-126 regulates the level of beclin-1 and improves cardiac function after acute myocardial infarction [129]. Beclin-1 is a specific mediator of autophagy that can be phosphorylated on Thr388 by AMPK [130], and its absent reduces excessive autophagy-induced cardiomyocyte death in the diabetic heart [131]. In addition, beclin-1 can be phosphorylated by unc51-like kinase1 (Ulk1) in response to amino acid deficiency and mTOR inhibition [132]. mTOR complex1 inhibits autophagy and regulates cell survival in brain injury and disease through the insulin/PI3K/Akt signaling pathway [133]. T2DM patients often have autophagy deficiency. Metformin increases autophagy flux in cardiomyocytes by activating SIRT1 or AMPK phosphorylation, contributing to cardio-protection [134]. On the contrary, antihyperglycemic medications through the insulin pathway decrease autophagy and exacerbate heart failure [134]. MiR-126-induced loss of IRS-1 suppresses glucose uptake, leading to energy deprivation, and its depletion can reduce autophagy by AMPK-dependent phosphorylation of Ulk1 [135].

Glycophagy is an alternative pathway for cytosolic glycogen storage and degradation. Starch Binding Domain 1 (STBD1) is linked to the Atg8 family member GABA Type A Receptor Associated Protein Like 1 (GABARAPL1) at the N-terminus and glycogen at the C-terminal CBM20 domain. This process subsequently stimulates glycogen phagocytosis and lysosome fusion, which leads to autophagosome maturation. Excess glycogen can then be removed from the lysosome by acid alpha-glucosidase (GAA) [136, 137]. Unlike the macrophagy, cardiomyocyte glycophagy occurs under the condition of insulin and exogenous high glucose necessarily accompanied by an increase in STBD1 expression [138]. Thus, as shown in Fig. 2, the reduction of miR-126 in diabetes myocardium is associated with autophagy and glycogen synthesis, which lead to glycogen pathology in the myocardium. So far, the regulatory mechanisms of exercises on glycogen accumulation and glycophagy are still inconclusive, and whether it is related to miR-126 needs further elucidation.

### Anti-apoptosis

Hyperglycemia causes the apoptosis of ECs and pancreatic β cells, aggravating the development of diabetic vascular complications [139]. MiR-126-5p inhibits cell apoptosis via its direct target protein TRAF7 which is one of the TRAF proteins involved in cell death and survival [32]. TRAF7 is a crucial regulatory protein that regulates whether the NF-κB transcription factor is activated or repressed [140]. TRAF7 also suppresses ECs apoptosis by increasing unusual polyubiquitination of the cellular fas-associated death domain-like IL-1-converting enzyme inhibitory protein (c-FLIP) [141] and the caspase-3-dependent pathway [142]. MiR-126-3p improves cell survival and decreases the expression of Bax and caspase-3 in high-glucose-induced human retinal ECs via targeting IL-17A and activating the PI3K/Akt pathway [143]. Due to the prevention of vascular apoptosis, exercise improves cardiovascular function. Aerobic exercise training significantly lowered blood pressure and heart rate in spontaneously hypertensive rats by increasing Bcl-2 levels [79]. Treadmill exercise promotes p-Akt expression, which assisted in the reduction of retinal apoptosis and neuron apoptosis in diabetic mice [144]. These findings imply that miR-126 and its downstream targets may be involved in the prevention of high glucose-induced apoptosis.

## Conclusions

Exercise is an important method for reducing cardiovascular risk and mortality rate in T2MD. MiR-126 can promote angiogenesis, decrease vascular inflammation, regulate autophagy and reduce endothelial apoptosis by targeting its downstream proteins. Endurance aerobic exercise with moderate-intensity or above can raise the level of circulating and myocardial miR-126 in diabetes, whereas RT need further investigation. Chronic aerobic exercise is preferred to acute ones for long-term advantages in the expressions of miR-126 and VEGF. MiR-126 is responsible for the beneficial effects of exercise and could be applied as an exercise indicator for cardiovascular prescriptions and as a preventive or therapeutic target for cardiovascular complications in T2DM.

## Data Availability

Not applicable.

## Abbreviations

T2DM: Type 2 diabetes mellitus
ECs: Endothelial cells
EPCs: Endothelial progenitor cells
SPRED1: Sprouty-related EVH1 domain-containing protein 1
PIK3R2: Phosphoinositide-3-kinase regulatory subunit 2
HMGB1: High-mobility group box 1
VCAM1: Vascular cell adhesion molecule 1
TRAF7: Tumor necrosis factor receptor-associated factor 7
CVD: Cardiovascular disease
ROS: Reactive oxygen species
m-miR-126: Myocardial miR-126
c-miR-126: Circulating miR-126
mu-miR-126: Muscle-miR-126
VSMCs: Vascular smooth muscle cells
IR: Insulin resistance
eNOS: Endothelial NO synthase
AGEs: Advanced glycation end products
GLUT4: Glucose transporter 4
IRS-1: Insulin receptor substrate-1
GSK3: Glycogen synthase kinase-3
KDR: Kinase insert domain-conjugating receptor
VEGF: Vascular endothelial growth factor
MVs: Microvesicles
EV: Extracellular vehicle
ERK: Extracellular signal-regulated kinase
SHR: Spontaneously hypertensive rats
MI: Myocardial infarction
STZ rats: Rats by streptozotocin injection
V̇O_2max_: Maximal oxygen consumption
HIIT: High intensity interval training
HVT: High volume training
MT: Moderate training
PPO: Peak power output
RT: Resistance training
CE: Chronic exercise
FG: Fasting blood glucose
Ang-1: Angiopoietin-1
Dlk1: Delta-like 1 homolog
NF-κB: Nuclear factor kappa B
TNF: Tumor necrosis factor
IL-1: Interleukin-1
IL-6: Interleukin-6
IKK: IκB kinase
SIRT1: Sirtuin 1
TLR: Toll-like receptors
RAGE: Advanced glycation end-products
AMPK: AMP activated protein kinase
Ulk1: Unc51-like kinase 1
mTOR: Mammalian target of rapamycin
STBD1: Starch Binding Domain 1
GABARAPL1: GABA Type A Receptor Associated Protein Like 1
GAA: Acid alpha-glucosidase
FoxO: Fork head box O
c-FLIP: Cellular fas-associated death domain-like IL-1-converting enzyme inhibitory protein
